# Avoiding fragmentation: The potential of synergistic efforts across the IMI portfolio

**DOI:** 10.3389/fneur.2022.1050360

**Published:** 2022-11-22

**Authors:** Carlos Díaz, Lewis Killin, Nigel Hughes

**Affiliations:** ^1^Synapse Research Management Partners SL, Madrid, Spain; ^2^Observational Health Data Analytics/Epidemiology, Janssen Research and Development, Beerse, Belgium

**Keywords:** neurodegeneration, public-private partnerships, cohort studies, research platforms, portfolio management, data standardization

## Introduction

There is a growing consensus in the scientific community that the harmonization and federation of data sources is a key enabler for the generation of actionable real-world evidence, which is essential to support timely decision-making. Healthcare systems, research communities, industry and, increasingly, citizens themselves generate data on a continuous basis. However, the wide range of methods used to capture, format, structure, and ultimately analyze such data limits the potential for data use and reuse. These obstacles are especially important when trying to solve research and clinical questions different from those that originally triggered data collection. Fostering transparent and efficient use of these data is essential for improving disease understanding and the development of much-needed novel therapies and interventions that can benefit the increasing number of patients affected by various degenerative, chronic, and debilitating neurological conditions worldwide.

## A fragmented and complex landscape of initiatives

Many previous efforts have focused on centralized data collection and processing, often within the context of single initiatives. These bespoke exercises, while beneficial, were frequently limited to the original research question, a local/regional/national focus, and/or time and funding considerations. Because of their custom nature, reusing collected datasets, data standardization pipelines, and derived tools is often too costly or technically difficult. New projects and studies usually resort to starting from scratch with their data collection and management strategies, which is not only inefficient but also causes delays and consumes valuable resources unnecessarily. In the research landscape, syndromes such as “reinventing the wheel” and “not invented here” are frequently visible.

In neurodegeneration (ND), for example, a plethora of specific cohort studies have been created stemming from individual memory clinics and clinical centers. These coexist with networks and global initiatives (e.g., the World Economic Forum's Davos Alzheimer's Collaborative),^1^ with different degrees of interaction among them. Most cohort studies collect broadly similar information, but they differ greatly in size, population, protocols, data formats, etc. The landscape is so varied and complex that specific efforts have been made to simply catalog existing cohorts and provide adequate metadata, such as in the Innovative Medicines Initiative (IMI) European Medical Information Network—(EMIF)^2^ project. Understanding what is available and under what conditions, as well as the potential for reuse, can be a daunting task.

Some current efforts go one step further, providing platforms for exploration, interrogation and, in some cases, aggregation or integration, as well as direct access to datasets, increasingly under federated models that respect the autonomy of contributing centers and alleviate concerns about ethical and legal issues associated with data protection. Examples include the Global Alzheimer's Association Interrogation Network (GAAIN),^3^ the EBRAINS Research Infrastructure,^4^ the Alzheimer's Disease Data Initiative (ADDI),^5^ and the recently launched, IMI-funded European Platform for Neurodegenerative Diseases (EPND).^6^ Most of these platforms offer several layers of access, allowing users to dig down from pure metadata browsing to actually performing analytics to varying degrees. Developing an enticing, ethically sound value proposition for researchers and data generators is critical for the ultimate success of these initiatives.

## Challenges and possible ways forward

Merely listing some of the current initiatives above demonstrates that fragmentation remains a major underlying issue in the ND field, and is likely one of the factors undermining the radical progress demanded by society for decades. The existence of a variety of solutions is not the problem – each is a valuable effort on its own – instead, the issue is that each new initiative is designed, developed, promoted, and attempted to be sustained in a practically isolated way. Ambition, innovation, outreach, buy-in from stakeholders, and true collaboration are all naturally limited beyond the confined space created by the specific funding flows supporting each endeavor.

Furthermore, as espoused by programmes such as IMI, public-private partnerships (PPPs) that include, but also support, multiple stakeholders in the public sector, e.g., research and enhancing clinical care, and the private sector, for research and development, have become an increasingly important model. In disease areas such as ND, PPPs can be an ideal framework to respond to this need due to the complexity of these diseases, the difficult nature of diagnostic and therapeutic development, and the required resources.

Switching from a maze of datasets and cohorts to a maze of platforms does not solve the current challenges in ND if the scientific richness and data generously contributed by citizens are constrained to one of the thousands of time-limited, insufficiently funded initiatives. Indeed, and given the scientific system structure and inertia, it does not seem that any given “definitive” solution will be able to resolve fragmentation on its own. Instead, it may be that more attention is needed toward key underlying issues such as: (1) data standardization and interoperability according to open standards in a transparent, agnostic, and flexible way; (2) programme management activities that are fully devoted to integrating individual projects and maximally exploiting synergies between them; and (3) system leadership approaches that promote open, non-judgemental spaces for peer-to-peer discussion and creativity across the range of stakeholder groups, enabling broad consensus on priority research questions to be tackled. The realization within research and clinical communities of the need for data harmonization has never been clearer than through the ongoing European Health Data and Evidence Network (EHDEN)^7^ project, an IMI-funded initiative that in the past 4 years has managed to mobilize over 250 healthcare and research institutions across Europe interested in mapping their data to the Observational Medical Outcomes Partnership (OMOP) Common Data Model. The use of an open common data model and derived analysis tools facilitates aggregated analyses of hundreds of millions of electronic healthcare records (EHRs) with speed, transparency, and privacy protection that can represent a true paradigm shift in the conduct of observational studies. By approaching standardization from a “research-question-agnostic” perspective, EHDEN is tackling a key challenge that hampers data use, and facilitating data being findable, accessible, interoperable, and reusable (FAIR). The impact of such innovative approaches can be seen also in the regulatory space, e.g., the recently launched DARWIN EU^®^^8^ initiative of the EMA.

Specifically, in the ND field, IMI has also been at the forefront of international data harmonization and integration efforts with regard to research cohorts, with flagship projects such as the aforementioned EMIF, the European Prevention of Alzheimer's Disease Consortium (EPAD),^9^ the Amyloid Imaging to Prevent Alzheimer's Disease project (AMYPAD),^10^ and the recently launched EPND. But importantly, this has been complemented at a higher, cross-project level, by the Efficiently Networking European Neurodegeneration Research (NEURONET)^11^ action, which has successfully built bridges across the IMI ND portfolio, creating a space where more than 20 distinct ND research projects could meet, discuss synergies, and generate new ideas. A key strength of NEURONET has been to support and communicate about the field neutrally and to remedy initiative fragmentation without constructing another new scientific hegemony. To that end, NEURONET has produced outputs that have represented the assets and experiences of its constituent studies as a transparent and one-stop resource. These are best represented by its Knowledge Base^12^ and its series of guidance deliverables, which outline cross-project experts' views and experiences on topics such as data sharing, data privacy, HTA and payer strategy, impact analysis, communication, and sustainability activities. The Knowledge Base in particular presents an accessible consolidation of resources that would otherwise be kept separate on an individual project or stakeholder channels. In addition, it enables the creation of tools of common interest that would not be in scope for any specific project. For example, the Regulatory and HTA Decision Tool signposts to different agencies, organizations, and case studies relevant to the assessment of new interventions, and the Asset Map graphically represents the usable outputs generated by any of the projects, ranging from disease models and ontologies to cohorts, datasets, and more. Importantly, this material is both applicable to the immediate IMI environment and to researchers who work outside of it. The privileged position of NEURONET as a neutral actor has also allowed it to organize meetings for “out-of-the-box” thinking, in an attempt to boost creative reflection around some of the most pressing research needs, without the limitations imposed by ordinary fora.

With its main role as a facilitator, NEURONET was well-positioned to establish the NEURO Cohort initiative. Here, it proposed a way of uniting 40 research and clinical sites across Europe, all interested in collecting a minimal data set about people living with or at risk of ND on a continuous basis in order to facilitate feasibility assessment and the establishment of future research projects. Critically, the design of the minimal dataset was done in collaboration with the sites to respect their autonomy whilst also reflecting their most commonly collected variables, which were also of interest to the community. Creating NEURO Cohort as an agreed baseline for common activity—with minimal overhead—has provided a foundation for further potential research at scale.

The “grassroots” approach of NEURONET, in which all projects and sites are equally important and participate on the same level in decision-making, can be seen as an initial template for the above-mentioned systems leadership philosophy, which can allow the gathering of stakeholders with differing interests – with none of them dictating the agenda – around a common objective.

## Conclusion

The acceleration of these coordination, harmonization, and integration efforts in recent years offers a unique opportunity to multiply and elevate concerted action to the next level, overcoming the inherent limitations of time-boxed and fixed-budget projects. This could also imply the creation of multi-stakeholder, sustainable observational spaces that go beyond data silos of specific types (e.g., EHRs, cohort data, patient-reported outcomes, and digital device data) or typical of certain research communities (e.g., clinical, regulatory, research cohort, and trial studies) to cut across them as well, at scale. A multi-project “Research Programme on Neuroscience” has recently been proposed that could link mapped EHR data from EHDEN (which captures medical history, drug use, co-morbidities, etc. of a large number of individuals) with the research cohort data from NEURONET (which capture deep phenotyping and biomarkers relevant to specific diseases and conditions, for a limited number of individuals). If successful, the data space resulting from synergies across two seemingly unrelated initiatives could become a unique resource attracting a variety of researchers, sponsors, and stakeholders, radically enhancing our global capacity for generating the necessary real-world evidence that can make a difference in addressing the ND diseases that affect millions.

For the past 15 years, IMI has been spearheading the creation of public–private consortia in Europe, involving hundreds of academic, healthcare, industrial, regulatory, and patient advocacy groups. It is important that the power of such a research ecosystem is not diminished by fragmentation and limitations resulting from project silos, and that appropriate action is taken to focus on key common challenges of global relevance, both within and outside the field of ND. This may necessitate new perspectives that promote programme management and integration as a priority. ND diseases represent a therapeutic area that clearly requires a collaborative research approach supported by considerable, relevant, and representative data, and this probably necessitates ambitious frameworks such as those developed by IMI's public–private partnerships. These, however, may need to be interconnected by design, as part of deeply integrated research programmes capable of mobilizing the capacity and resources required to provide faster and more efficient progress in the field.

## Author contributions

CD, LK, and NH wrote sections of the manuscript. All authors contributed to manuscript revision, read, and approved the submitted version.

## Funding

This work was supported by funding from the Innovative Medicines Initiative 2 Joint Undertaking (JU) under grant agreement number 821513 (NEURONET) and 806968 (EHDEN). The IMI JU receives support from the European Union's Horizon 2020 research and innovation programme, EFPIA, and the Parkinson's Disease Society of the UK LBG.

## Conflict of interest

Author NH was employed by Janssen Pharmaceutica NV. Authors CD and LK were employed by Synapse Research Management Partners SL.

## Publisher's note

All claims expressed in this article are solely those of the authors and do not necessarily represent those of their affiliated organizations, or those of the publisher, the editors and the reviewers. Any product that may be evaluated in this article, or claim that may be made by its manufacturer, is not guaranteed or endorsed by the publisher.

## Footnotes

1 www.davosalzheimerscollaborative.org

2 www.emif.eu

3 www.gaain.org

4 www.ebrains.eu

5 www.alzheimersdata.org

6 www.epnd.org

7 www.ehden.eu

8 www.ema.europa.eu/en/about-us/how-we-work/big-data/data-analysis-real-world-interrogation-network-darwin-eu

9 www.ep-ad.org

10 www.amypad.eu

11 www.imi-neuronet.org

12 https://kb.imi-neuronet.org

